# Factors associated with intimate partner physical violence among women attending antenatal care in Shire Endaselassie town, Tigray, northern Ethiopia: a cross-sectional study, July 2015

**DOI:** 10.1186/s12978-017-0337-y

**Published:** 2017-06-24

**Authors:** Berhane Hailu Gebrezgi, Marta Berta Badi, Endashaw Admassu Cherkose, Negassie Berhe Weldehaweria

**Affiliations:** 1 College of Health Sciences, Department of Midwifery, Aksum University, P.O.Box-1010m, Aksum, Ethiopia; 2 College of Health Sciences, Department of Midwifery, University of Gondar, Gondar, Ethiopia; 3 College of Health Sciences, Department of Public Health, Aksum University, Aksum, Ethiopia

**Keywords:** Intimate partner physical violence, Pregnancy, Antenatal care, Tigray, Ethiopia

## Abstract

**Background:**

Intimate partner physical violence is a common global phenomenon. About 30.00% and 38.83% of women in the world and in sub-Saharan Africa experienced physical violence by their partner respectively in 2013. Though intimate partner violence has serious adverse health consequences, there is limited information about partner violence during pregnancy in Ethiopia. Therefore, the aim of this study was to assess the prevalnce of physical intimate partner violence during pregnancy and associated factors among women attending antenatal care in Shire Endaselassie town, Tigray, northen Ethiopia

**Methods:**

A facility based cross-sectional study was conducted from May 3 to July 6, 2015. Four hundred and twenty-two pregnant women attending three public health facilities were included using systematic sampling technique. In addition, twenty-two purposely selected key informants were interviewed. The data collectors and supervisors were trained on all data collection processes. Data were entered to Epi-Info version 7.1.2.00 and exported to SPSS version 20.00. Logistic regression was used to identify factors associated with intimate partner physical violence. Statistical significance was declared at *p* < 0.05. Qualitative data were categorized into themes and triangulated with the quantitative results.

**Results:**

The prevalence of intimate partner physical violence in pregnancy was 20.6% (CI = 16.70, 24.90). Age at first marriage greater than or equal to 17 years (AOR = 4.42, CI = 2.07, 9.42), women with no formal education (AOR = 2.78 CI = 1.10, 7.08), rural dwellers (AOR = 2.63 CI = 1.24, 5.58), intimate partners with no formal education (AOR = 2.78 CI = 1.10, 7.08) and intimate partner alcohol consumption (AOR = 3.8 CI = 1.85, 7.82) were factors associated with intimate partner physical violence towards pregnant women.

**Conclusion:**

Nearly one fifth of women surveyed experienced intimate partner physical violence during pregnancy. Early marriage, rural dwelling, intimate partner alcohol consumption, and educational status were associated with intimate partner physical violence during pregnancy. Urgent attention to women’s rights and health is essential at all levels to alleviate the problem and its risk factors in Tigray regional state of Ethiopia.

## Plain English summary

Intimate partner physical violence against pregnant women is common globally and in sub-Saharan Africa. Violence during pregnancy has serious negative health consequences for both mother and fetus. This study assessed prevalence of physical violence during pregnancy and associated factors among women attending antenatal care in Shire Endaselassie town, Tigray, northern Ethiopia.

**Fig. 1:**
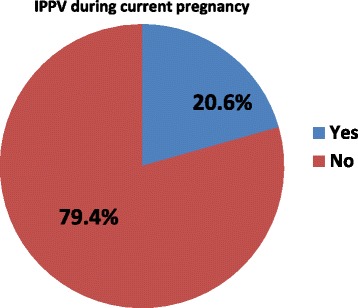
Prevalence of intimate partner physical violence among pregnant women attending antenatal care services in public health facilities in Shire Endaselassie town, northern Ethiopia, 2015

**Table 1:** Socio-demographic characteristics of pregnant women attending antenatal care services in public health facilities in Shire Endaselassie town, northern Ethiopia, 2015

Variables	Frequency	Percent
Age of women		
15–24	130	30.80
25–34	226	53.60
> 34	66	15.60
Marital status		
Single	6	1.40
Married	404	95.50
Divorced	7	1.70
Widowed	5	1.20
Religion		
Orthodox	380	90
Muslim	42	9.80
Ethnicity		
Tigray	403	95.50
Amhara	19	4.50
Women educational status		
No literate	136	32.20
Read & write	57	13.50
Elementary	98	23.30
High school	91	21.60
Collage & above	40	9.40
Intimate partner educational status		
No literate	103	24.40
Read & write	15	3.60
Elementary	122	28.90
High school	127	30.10
Collage & above	55	13
Women occupation		
House wife	256	60.70
Government employee	26	6.20
Private work	140	33.10
Intimate partner occupation		
Farmer	102	24.20
Government employee	69	16.40
Private work	251	59.50
Women monthly income(ETB)		
< 500	228	54
500–1000	104	24.60
> 1000	90	21.30
Intimate partner monthly income (ETB)		
< 500	13	3.10
500–1000	119	28.20
> 1000	290	68.70
Residence		
Urban	307	72.70
Rural	115	27.30
Intimate partner alcohol consumption in a week		
Yes	141	33.40
No	281	66.60

**Table 2:** Reproductive characteristics of pregnant women during pregnancy among pregnant women attending antenatal care services in public health facilities in Shire Endaselassie town, northern Ethiopia, 2015

Variables	Frequency	Percent
Age at marriage (in years)		
< 18	160	37.90
≥ 18	262	62.10
Gravidity		
Primi gravid	147	34.80
Multi gravid	275	65.20
Parity		
Primi parity	104	24.6
Multi parity	318	75.4
Who choose her intimate partner		
Family	118	28
Herself	304	72

**Table 3:** History of PV and related characteristics among pregnant women attending antenatal care services in public health facilities in Shire Endaselassie town, northern Ethiopia, 2015

Variables	Frequency	Percent
Reported history of PV among pregnant women’s mothers (*n* = 422)		
Yes	101	23.95
No	290	68.70
Do not know	31	7.30
Dominance in decision making of household affairs (*n* = 442)		
Equally	310	73.5
Husband	104	24.6
Wife	8	1.9
Decision making in purchasing household materials (*n* = 422)		
Equally	331	78.40
Intimate partner only	84	19.90
Wife only	7	1.70
Intimate partner support during pregnancy (*n* = 422)		
Yes	417	98.80
No	5	1.20
Frequency of partner alcohol consumption (*n* = 142)		
Consume every day	37	26.06
Consume 1–2 times a week	48	33.80
Consume 1–3 times a month	57	40.14
Reported reasons of wife beating (102 with multiple response)		
If wife disobey	95	93.14
If wife did not complete housework	91	89.22
If wife refuses sex	82	80.39
If wife is unfaithful	74	72.55
If wife asks intimate partner whether he has other wife	71	69.61
Reasons of perpetration (*n* = 87)		
Husband consume alcohol	39	44.83
When wife disobey or argued with husband	24	27.59
When wife refuse to have sex	14	16.09
When wife did not complete housework	10	11.49
Magnitude of PV during pregnancy compared with non-pregnancy time (*n* = 87)		
Get worsen	14	16.09
Lesser	10	11.50
The same	63	72.41
Frequency PV during current pregnancy (*n* = 87)		
Once	24	27.59
Twice	37	42.53
More than twice	26	29.89

**Table 4:** Type of physical violence during pregnancy among pregnant women attending antenatal care services in public health facilities in Shire Endaselassie town, northern Ethiopia, 2015

Type of physical violence	In current pregnancy		In past pregnancy	
	Yes	No	Yes	No
	Freq (%)	Freq (%)	Freq (%)	Freq (%)
Slapped or thrown something, beating	74(85.10%)	13(14.90%)	55(87.30%)	8(12.70%)
Pushed, shoved or pulled her hair	73(83.90%)	14(16.10%)	51(81.00%)	12(19.00%)
Choked on purpose	39(44.80%)	48(55.20%)	41(65.10%)	22(34.90%)
Threatened to use or actually used a gun, knife or other weapon	32(36.20%)	55(63.20%)	15(23.00%)	48(76.20%)

**Table 5:** Factors associated with intimate partner physical violence among pregnant women attending antenatal care services in public health facilities in Shire Endaselassie town, northern Ethiopia, 2015

Variables	IPPV during pregnancy		COR (95% CI)	AOR (95% CI)
	Yes	No		
Age at marriage				
< 18	72	88	13.47[7.34,24.72]	4.42[2.07, 9.42]***
≥ 18	15	247	1	1
Gravidity				
Primi gravid	9	83		
Multi gravid	78	252	2.85[1.37,5.94]	1.58[0.23,1.72]
Women’s Educational status				
No formal education	68	126	13.6[5.3,34.88]	3.40[1.11,10.42]*
Elementary	14	84	4.16[1.44,12]	3.05[0.88,10.55]
High school & above	5	125	1	1
Women’s occupation				
House wife	77	179	6.7[3.36,13.42]	1.06[0.41,2.75]
Private & Gov’t Employed	10	156	1	1
Residence				
Rural	55	60	7.85[4.70,13.21]	2.63[1.24,5.58]**
Urban	32	275	1	1
Intimate partner’s age				
20–34	18	157	1	1
35–44	47	135	3.04[1.68,5.48]	1.01[0.39,2.44]
≥ 45	22	43	4.46[2.20,9.06]	1.02[0.32,3.00
Intimate partner education				
No formal education	59	59	15.54[7.66,31.57]	2.78[1.10,7.08]*
Elementary	17	105	2.51[1.14,5.57]	1.86[0.69,4.97]
High school & above	11	171	1	1
Intimate partner alcohol use				
User	76	66	10.71[1.16,18.63]	3.80[1.85,7.82]***
None user	21	259	1	1
Childhood PV				
Experienced	59	45	13.58[7.85,23.50]	3.94[2.26,10.81]
Not experienced	28	290	1	1
Women’s Attitude towards IPPV				
Positive attitude	40	40	6.27[3.67,10.72]	1.07[0.21, 1.00]
Negative attitude	47	295	1	1

*** *p* < 0.001, **p < 0.01, *p < 0.05

A written survey was collected at three public health facilities to obtain information related to intimate partner physical violence for 422 pregnant women. In addition, 22 women were interviewed. The data collectors and supervisors were trained on relevant methods. Analysis was conducted to identify factors associated with intimate partner physical violence in pregnancy. Interview data were categorized into themes and compared with the survey results.

About one-fifth of women experienced physical violence in pregnancy. Early marriage, rural dwelling, intimate partner alcohol consumption, and educational status were associated with a higher prevalence of physical violence during pregnancy. Urgent attention is essential at all levels to alleviate the problem of physical violence and its risk factors.

## Background

The World Health Organization (WHO) defines intimate partner violence as one of the most common forms of violence against women and includes physical, sexual, emotional abuse and controlling behaviors by an intimate partner [1]. Intimate partner physical violence (IPPV) is more common than some health conditions routinely screened for in antenatal care [2].

Worldwide, about 35.00% of women experienced physical violence committed by their intimate partners [1]. Intimate partner physical violence is highest in sub-Saharan Africa, where 38.83% of women were abused by their intimate partners [3]; the prevalence in Nigeria, Rwanda and Tanzania was 22.90%, 19.30% and 18.00% respectively [4–6]. In Ethiopia, intimate partner violence was reported as high as 64.70% [7] and 76.50% [8]. A study in Abay Chomen district, Western Ethiopia revealed 44.50% women experienced intimate partner violence in a recent pregnancy [9]. This higher prevalence may be due to women’s disadvantaged position in the country’s patriarchal society [9]. The Ethiopian Demographic Health Survey (EDHS) report in 2011 demonstrated that physical violence against women was common, in both urban and rural areas. In addition, according to the EDHS report, 68% of women agree that wife beating is justified [10].

Intimate partner violence during pregnancy has been associated with fatal and non-fatal adverse health outcomes due to the trauma and stress of abuse [11]. There is particular concern if the abdomen is targeted during pregnancy due to the risk of injury to the fetus [11]. Violence in pregnancy adds to the many challenges such as unintended pregnancies, pregnancy-related distress, inadequate prenatal care, induced and spontaneous abortion, gestational weight gain, intrauterine restriction, hypertension, pre-eclampsia, third trimester bleeding, sexually transmitted infections (STIs) and risks of death [9, 12–15]. Neonatal and postnatal death are increased twofold and threefold respectively, among women suffering physical violence by their husband [4, 12, 13].

In Ethiopia, studies among non-pregnant women, indicate physical violence against women is common [16, 17]. Though physical violence during pregnancy has significant health risks to the mother and her fetus, there is limited information about the prevalence and risk factors of intimate partner physical violence during pregnancy. Therefore, this study assessed the prevalence and factors associated with intimate partner physical violence during pregnancy in northern Ethiopia.

## Methods

### Study area and period

The study was conducted in Shire Endaselassie town, the administrative town of Northwest zone, Tigray region, northern Ethiopia. Shire Endaselassie town is the third most populated town in the region. It is located 1087 km north of Addis Ababa, the capital city of Ethiopia, and 304 km northwest of Mekelle, the capital city of Tigray. Based on the 2007 Ethiopian Central Statistics Agency report, this town has a total population of 47,284, of whom 21,867 are men and 25,417 women [18]. The town has one general hospital, two governmental health centers and four private clinics. All the three public health facilities and one private clinic were providing antenatal care (ANC). The study was conducted in one general hospital and two health centers from May 3 to July 6, 2015.

### Study design

Facility based cross-sectional study was triangulated by qualitative data.

### Source and study population

The study population was pregnant women who attended ANC in selected public health facilities during the study period and who resided in Shire Endaselassie town for at least six months. Pregnant women who were critically ill and who are unable to respond were excluded.

### Sample size calculations

The required sample size was determined using single-population proportion with the following assumption: 50% prevalence of intimate partner physical violence during pregnancy in Ethiopia, since there has been no study done on this specific topic in Ethiopia. Assuming 95% of confidence interval, 5% marginal error and adding 10% for non-response rate; the final sample size was 422.

### Sampling technique and procedures

Participants were selected using systematic sampling, the constant K was calculated for public health facilities using the formula K = N/*n* = 2400/422 = 5. Every fifth pregnant woman visiting the public antenatal clinics was approached. Pregnant women who attended ANC clinics for an average of two months in the previous year were used to estimate recruitment from each health facility. The average client flow in the months of May to July 2013 at each facility was; 604 in Hospital Suhul, 996 in Alganesh health, and 800 in Oumore health center. The sample was derived proportionately based on the previous year’s client flow. For the qualitative part of the study, 22 key informants were selected purposefully. The criteria to select interview participants were pregnant women with the ability to express the required information about the problem clearly.

### Study variables

#### Dependent variable

Intimate partner physical violence during the current pregnancy.

#### Independent variables

Individual variables; age, religion, educational status, residence, gravida, history of violence in childhood, alcohol use, acceptance of violence against women, male dominance in family affairs, monthly income, polygamous relationship, early marriage, decision-making power.

Societal factors; gender-inequality, social and economic status of pregnant women, weak community sanctions against IPPV, social acceptance of violence against women [16].

### Operational definitions

#### Intimate partner physical violence during pregnancy

Intimate partner physical violence was defined as the intentional use of physical force towards pregnant women with the purpose of causing death, disability, injury, or harm. Physical violence included, but was not limited to, scratching, pushing, shoving, throwing, grabbing, biting, choking, shaking, slapping, punching, burning, or use of a weapon [1].

#### Intimate partners

Intimate partners included current and former common-law partners, non-marital partners, and marital partners [19].

### Data collection

#### Quantitative data

A pretested semi-structured locally adapted questionnaire, based on the WHO Multi-country Study on Women’s Health and Life Experiences [16], was used. The questionnaire was prepared in English, and then translated to Tigrigna (local language) and back to English to maintain consistency of the tool. Translations were done by two midwives from Aksum University with good knowledge of the local language.

#### Qualitative data

Face-to-face in-depth interviews were conducted to explore how pregnant women experienced physical intimate partner violence. During the interview, topics explored included the causes and types of violence, community norms regarding IPPV and their experience of previous IPPV. A total of 22 key informants (14 pregnant women and eight from the women’s affairs office) were interviewed until saturation was reached. Audio recordings and handwritten notes were taken at the time of the interview by an experienced interviewer. Two experienced masters’ holders interviewed key informants. The average length of interviews was thirty minutes.

#### Data quality control

The questionnaire and interview guide was pretested in Aksum town among 21 women visiting public antenatal clinics (one hospital and one health center). Training was provided for three female midwives for questionnaire administration, two MPH qualitative data collectors and one supervisor were recruited and trained on how to collect and supervise data collection. The trained data collectors were supervised during data collection, and each questionnaire was checked for completeness. The principal investigator and supervisors re-administered 5% of all questionnaires to confirm their validity. Data entry was conducted by two different individuals at two different computers to minimize error, and the sameness of data entered was checked. The correctly entered data were used as the final data. Two experienced interviewers conducted key informant interviews, and one qualified transcriber was used for all qualitative data.

### Data processing and analysis

#### Quantitative data

After the data were checked for completeness and accuracy, it was coded, entered to Epi Info version 7.1.2.0 and exported to SPSS version 20.00 for analysis. Bivariate analysis between dependent and independent variables was performed using binary logistic regression. To select variables for the multivariate analysis a *p*-value <0.20 was used. To adjust for confounding variables, a multivariable logistic regression was done, and a *p*-value <0.05 with 95% confidence interval (CI) for odds ratio (OR) was used to determine significance.

#### Qualitative data

Qualitative data was transcribed from the recorded audio and notes to the local language (Tigrigna) and translated to English narratives. After repeated review of translated narratives, the transcripts were reduced, coded and similar ideas were grouped together into themes manually. Finally, ideas were triangulated with the quantitative results.

### Ethical considerations

Ethical clearance was obtained from the University of Gondar College of Medicine and Health Sciences Ethical Review Board (IRB). A formal letter of study approval was obtained from Shire Endaselassie town health office and Tigray regional health bureau. After securing necessary permissions, written informed consent was obtained from all participants. For pregnant women under 18, written informed consent was secured considering as mature minors to secure participants’ privacy [20, 21].

## Results

### Socio-demographic characteristics of the pregnant women

Four hundred twenty-two pregnant women were involved in this study, yielding a response rate of 100%. More than half of the pregnant women (226; 53.60%) were 25–34 years, and the mean age was 27.98 years (SD ± 6.30). The majority of pregnant women (380; 90%) were Orthodox Christian and 37 (8.85%) were Muslim. More than nine in ten, of the pregnant women (404; 95.70%) were married and 403 (95.50%) were Tigray in ethnicity. Regarding their educational status, 136 (32.20%) pregnant women had no formal education (Table 1).

Nearly three quarters, of the pregnant women (256; 60.70%) reported their occupational status was housewife. About two thirds of the pregnant women (307; 72.70%) were urban dwellers. One hundred eighty-three (43.40%) of the intimate partners were 31–40 years. About one third (30.10%) of the partners were high school graduates. More than two thirds of the pregnant women (290; 68.70%) reported their intimate partner’s monthly income was greater than 1000 ETB (45.50 USD). About one third, 141 (33.60%) of partners were alcohol consumers (Table 1).

### Reproductive characteristics of pregnant women

The mean age at marriage of participants was 17.11 (SD ± 3.18) years. The mean gravidity and parity of respondents were 2.9 (SD ± 1.89) and 1.8 (SD ± 1.60) respectively. Nearly one third of pregnant women (118; 28%) reported that their intimate partner was selected by their family (Table 2).

### History of physical violence and related factors

Among the pregnant women, 101 (23.95%) reported that their mother had been beaten by their intimate partner (Table 3). Three hundred and ten (73.50%) pregnant women reported that they jointly managed their household with their intimate partner; however, 104 (24.60%) reported that they were overruled by their intimate partners in managing household affairs. Over three quarters (78.40%) of pregnant women reported they participated equally in household purchasing. Almost all, 417 (98.80%) of women, stated that their intimate partner supported their pregnancy (Table 3). A 21-year-old gravida 2 woman said that *... when I was a 7 months pregnant my husband sold our ox without my will. Then, I asked him why he did that. He responded that ‘I am the head of the family. I can do whatever I like’. When I tried to convince him, he choked me and I started to bleed through my mouth and I lost one tooth.*

Greater than three quarters of participants (76; 87.35%) reported that perpetrators of violence were not restricted to participate in social events like informal community leadership. According to pregnant women who experienced IPPV, 44.83% experienced violence when their partner consumed alcohol (Table 3). A key informant from the women’s affairs office stated that *...starting from the day of marriage ceremony there is an indicator of accepting wife beating (laughing)...the family of the wife selects one person from the intimate partner’s relatives, called wahas (guarantor). When the intimate partner beats his wife, she may go to guarantor and then he convinced her to accept an intimate partner beating and then to continue their relationship with her partner. Due to this reason, many women keep their secret of intimate partner beating and they didn’t come to us.*

There were concerning explanations for violence, about one quarter 102 (24.20%) reported that their intimate partner has the right to beat them for at least one justified reason (Table 3). In support of this, a 36-year-old housewife gravida 5 who experienced intimate partner physical violence said that *...One day when I was about 5 months pregnant, my husband beats me by stick on my back and thighs. The reason was because I leave the house alone and went to the market.*

About seven in ten (72.41%) reported that the magnitude of violence was unchanged during pregnancy, while fourteen (16.09%) women stated that the magnitude increased and 10 (11.50%) stated it decreased during pregnancy. Thirty-seven (42.53%) and 26 (29.89%) of the pregnant mothers faced PV two times and more than two times when they were pregnant respectively. About one quarter (27.59%) of the women experienced PV only once during the current pregnancy (Table 3).

### Prevalence of intimate partner physical violence during current pregnancy

The prevalence of IPPV during the current pregnancy was 20.6% (CI = 16.70, 24.90) (Fig. 1). For those who experienced IPPV in the current pregnancy, 74 (85.10%) were slapped or had items thrown at them and thirty-nine (44.80%) were choked by their intimate partners (Table 4).

Twenty pregnant women (22.98%) who faced PV during their current pregnancy reported that they were kicked in the abdomen. A 30-year-old gravid 3 woman said*...during my first pregnancy one day my intimate partner drank a lot of “swa” (a local alcohol) and he kicked me in my abdomen two times. Then, immediately after I started to bleed through my vagina. After three days of bleeding, I delivered a dead baby in my home (crying).*

### Factors associated with IPPV during the current pregnancy

The binary logistic regression showed that age at marriage, gravidity, women’s educational status, women’s occupation, residence, intimate partner’s age, intimate partner’s education, intimate partner’s alcohol consumption, childhood PV and women’s attitude towards IPPV were associated with IPPV in the current pregnancy. In the multivariable analysis; age at marriage, women’s educational level, residence, intimate partners’ educational level and intimate partner’s alcohol consumption were significantly associated with IPPV during the current pregnancy. The Hosmer-Lemenshow goodness of fit test (*p = 0.415*) provides evidence of model fit with the predictor. Pregnant women who were married at 18 or younger were 4.42 times more likely to experience IPPV during their current pregnancy (AOR 4.42, 95% CI = 2.07, 9.42) (Table 5). A 31-year-old gravid 3 woman pointed that *...The abuse was started immediately when I got married. I was hitsankolea (to mean small child)... he also beat me when I was pregnant.*

Women with no formal education were 3.40 times more likely to face IPPV during pregnancy than those with high school education or greater (AOR = 3.40, 95% CI = 1.11, 10.42). Pregnant women from rural areas were 2.63 times more likely to experience PV by their intimate partners (AOR = 2.63, CI = 1.24, 5.58) (Table 5). A 21-year-old gravid 2 woman said that *...most intimate partners at this time did not beat their wives ... but some numbers of housewives are not as such informed about their rights, mostly those found in the rural areas when compared with urban dwellers.*

Partners with no formal education were 2.78 times more likely to physically assault their wives (AOR = 2.87, CI = 1.10, 7.08) (Table 5). A 34-year-old gravid 5 mother reported that *...until this time uneducated intimate partners...Ayashu Sewut (to mean foolish husbands) beat their wives. But most of the educated partners did not beat their wives.*

Pregnant women whose intimate partners consumed alcohol were 3.80 times more likely to experience PV (AOR = 3.80, CI = 1.85, 7.82) (Table 5). A forty-year-old gravid 7 woman who experienced IPPV said that *....my intimate partner beats me many times when he was drunk... he aggressively starts to insult me and my children. One time at night, he came home after he drank at a wedding, he beat me with a big log on my legs and all around my body… I mean he wanted to kill me.*

## Discussion

The prevalence of IPPV during pregnancy in this study was 20.60%. This is consistent with studies in other African countries, Nigeria, Rwanda and Tanzania, which showed a prevalence of 22.90%, 19.30% and 18% respectively [4–6]. Furthermore, these findings are supported by a study conducted in India, which states that the prevalence of IPPV during pregnancy was 23% [22]. However, it is lower than the community based study in Western Ethiopia among married pregnant women, which reported the prevalence of IPPV is 29.2% [9]. This difference might be due to difference in study designs, as the study conducted western Ethiopia was community based study [9] whereas this study was health facility based which may miss those women who were not coming to health facilities. The difference in IPPV rates between regions might be due to differences in community perceptions towards IPPV. The high prevalence of IPPV may lead to poor pregnancy outcome and maternal psychological and physical health problems [23].

Twenty women (22.98%) who faced PV during pregnancy, were kicked in their abdomen. This is in line with a multi-country study which shows between one quarter and one half of the pregnant victims were kicked in the abdomen [16]. Furthermore, a study in Bangladesh showed a 37% of urban and 25% of rural pregnant women were kicked in their abdomen while they were pregnant [24]. It is also supported by the qualitative data where some of the pregnant women stated that perpetrators were kicking them in the abdomen, which is life threatening for the mother and the fetus.

About one quarter of the pregnant women in this study believed that men have the right to beat their wives during pregnancy**.** A study conducted in western Ethiopia, reports a similar finding that women were expected to be tolerant [8]. This result was also supported by the qualitative data. Some women from the women affairs office claimed that many hide violence and they did not inform the police or any other legal body. That might be due to socio-cultural dynamics which encourage women to be tolerant.

Women who were married at less than 18 years were 4.42 times more likely to experience violence by their intimate partners during pregnancy. Similarly, the Demographic Health Surveys in Kenya, Bangladesh, Bolivia, Rwanda, and Zimbabwe showed that women who married at younger ages were more likely to experience PV [25]. The 2011 EDHS revealed that domestic violence in Ethiopia was justified if the husband was not satisfied by his wife’s cooking, due to arguments, if she went out without permission, if he perceived her to neglect the children, or the refusal of sexual advances [10].

In this study, women who had no formal education were 3.40 times more likely to experience IPPV during pregnancy than those with a high school education or greater. Studies in Tanzania and Rwanda also showed pregnant women with no formal education were more likely to experience violence by their intimate partners [4, 6]. This is also consistent with a study in Nigeria which showed pregnant women with no formal education were at 2.43 times increased risk of IPPV compared to those who have formal education [26]. Likewise, a study done in Iran showed that pregnant women who have no formal education were more likely to experience PV [27]. This may be due to the fact that pregnant women with no formal education have less access to information towards women empowerment or they may have more acceptances for PV than educated women [28].

Pregnant women whose intimate partners had no formal education were 2.78 times more likely to abuse their wives than those who had a high school education or greater. In contrast, a study done in Nigeria stated that intimate partners who had no formal education had a lower prevalence of PV [26]. This may reflect socio-cultural differences such as high acceptance of wife beating in Ethiopia. A study done in Bangladesh showed that pregnant women whose intimate partners had more than 10 years of schooling had a lower odds of experiencing PV by their intimate partners during pregnancy [24]. This might be due to the fact that partners with no formal education were more likely to have traditional perceptions regarding gender equality [24]. One pregnant woman supported this idea by saying *most of the intimate partners who beat their wives are uneducated and account traditional belief of wife beating as a norm*.

In this study, rural dwellers were 2.23 times more likely to experience PV by their intimate partners as compared to those living in urban areas. This is supported by a study conducted in Bangladesh which found that pregnant women from rural areas were more likely to experience PV than those from urban settings [24]. One pregnant woman stated that *most of the perpetrators are in the rural area where victims are not aware of their equality right with their intimate partners and are more influenced by traditional influences*.

Pregnant women, whose intimate partners consumed alcohol, were 3.80 times more likely to experience IPPV. This finding is consistent with those from Kenya, Zambia and Bolivia on IPPV which reported alcohol consumers abused their wives more frequently [25]. Other studies from Brazil [29] and Turkey [30] also revealed alcohol consumers were more likely to violate their wives. Some victims of PV described that their intimate partners beat them when they consumed excessive alcohol. This might be due to the fact that when they consumed alcohol, they may have been more likely to become aggressive [31, 32].

### Limitations of the study

Cross-sectional studies, such as this, cannot identify causation. In addition, since intimate partner violence is a sensitive issue, some participants may chose not to disclose and this would lead to underreporting.

## Conclusion

One fifth of pregnant women experienced intimate partner physical violence in their current pregnancy. Some of the pregnant women who experienced IPPV reported that they were kicked in the abdomen while pregnant. Age at first marriage less than 18, women’s education, residence, intimate partner’s education and alcohol consumption were factors associated with intimate partner PV during pregnancy. One quarter of pregnant women believed that intimate partners have the right to beat their wives even when pregnant.

### Recommendation

Based on these findings, we recommend Tigray regional health bureau design and implement programs and interventions jointly with antenatal care services to lower IPPV. IPPV screening should be conducted by health facilities during antenatal care as the best window to address violence against women in pregnancy. Furthermore, health extension workers and women development armies (women’s networks) should be engaged in education, screening and referral of IPPV victims to health facilities. Further longitudinal studies are recommended to strengthen the evidence about magnitude and associated factors of IPPV.

## Abbreviations

ANC: Antenatal care
AOR: Adjusted odds ratio
CI: Confidence interval
COR: Crude odds ratio
EDHS: Ethiopian Demographic Health Survey
ETB: Ethiopian birr
IPPV: Intimate partner physical violence
IRB: Institutional review board
MPH: Master of Public Health
OR: Odds ratio
PV: Physical violence
SD: Standard deviation
STIs: Sexually transmitted infections
WHO: World Health Organization
